# Progress in Psychiatry

**Published:** 1900-08-04

**Authors:** 


### Progress in Psychiatry.

(*Continued from page* 188.)

**Genius and Degeneracy.**—Our knowledge of human psychology has been greatly enriched by pathological facts. Since mental diseases have begun to be systematically and scientifically studied a transformation has been brought about in the science of normal psychology. Psychiatry and psychology are to-day closely and indissolubly united, and the bond could only be severed to the detriment of both. In recent years psychologists have bestowed considerable attention on the study of the subject of genius in its various aspects. Various and even contradictory conclusions as to the nature and constitution of genius have been reached by different investigators. Unfortunately, much confusion has been created by regarding genius as a variety of insanity, and this at a time when the conceptions alike of insanity and of genius were not as yet scientifically formulated. Some authors would call a person endowed with especially excellent mental powers a genius. Wieland[38] speaks of three kinds of genius: "The genius of pleasing, which operates within the domain of the graces, and consists in a special facility for carrying out the idea it prosecutes; philosophical genius, which consists in a capacity for discovering those truths resulting from correct views concerning the happiness of mankind; and practical genius, which consists in a ready activity in availing itself of known facts and in producing the highest and most prompt resolution." It will be conceded that this is a somewhat arbitrary if convenient description and division of the subject. H. Joly[39] says: "Genius is a creative power, using the term in the relative sense in which alone it is permissible to apply it. It is the production of something which the combined efforts of other men have been powerless to effect. It is that which puts at the disposal of humanity means of expression, of talent, of invention, or of new action, which add something to the stock of common intelligence and common power." Hagen would connote as the chief characteristic of genius "high originality of conception, of discovery, and of creative impulse." Originality, *per se*, does not constitute genius, for many a crank and faddist with original ideas is far from being a genius. The person who writes a book intended to convince society that men and women should go naked is a crank, not a genius. The modern troglodyte[40] referred to in a contemporary comes under the same category. The man who publishes far and wide that he could cure all ills that flesh is heir to by drinking water, or by making people walk barefooted over wet grass, is scarcely a genius. The faith-healer and Christian scientist lacks originality, and his claims are equally doubtful. The people might gape and regard with awe his pretensions, but his temporary success in working on the "eternal gullible" would succumb inevitably in the end before scientific investigation. The genius for poetry and music is not the same as that for science and philosophy, for the physical conditions are not identical. Yet the almost universal attestation of the genius of Descartes, Galileo, and Newton in philosophy and mathematics; of Shakespeare and Goethe in literature; of Mozart and Wagner in music; of Darwin and Herbert Spencer in the biological sciences; shows that the underlying quality of genius gives to many and different persons its special hall-mark. Genius may be exhibited in other spheres of action. Thus, Frederick the Great was a political and military genius, Napoleon Bonaparte a military and ruling genius, Bismarck a statesman of genius.

Moreau de Tours,[41] a physician and psychologist of eminence, considered genius as a semi-morbid condition resulting from over-excitation of the brain. "Genius, like every other intellectual condition of dynamism, has its substratum . . . and this substratum is a semi-morbid state of the brain, a true nervous erethism. . . . Whenever the psychical forces surpass the ordinary limits of their action . . . the cause must be sought in certain neuropathic conditions of the subject." This neuropathic element, regarded as a constituent part of genius, is insisted upon by Lombroso, who in his classical work[42] has laboriously collected numerous instances to illustrate it. Yet there can be little doubt concerning the perfect sanity of such men of genius as Shakespeare and Goethe, Kant, Darwin, and Herbert Spencer. Temporary or periodic sensorial disturbances, either in the form of illusions or hallucinations of sight or hearing, have been not uncommonly met with in men of genius, and among the names of such men collected by Moreau and Lombroso are those of Socrates, Martin Luther, Benvenuto Cellini, Cromwell, Bernadotte, and Byron. Resemblances to the impulses of monomania or to the emotional susceptibilities and imaginings of hysteria may be traced in men of genius, but it may be questioned whether such conditions are affinities or mere superficial resemblances. The trained psychologist and the practical physician will distinguish without much difficulty between changes of mood such as Goethe observed in himself—and which have their origin in the highest developments of the psychical organism — and the capriciousness of a hysterical woman at whose bidding there stands always at need a rich well of tears or a rich fount of morbid sentiment and romance whose taps have but to be turned to allow of a ceaseless flow. Insanity has occasionally attacked great men. Jean Jaques Rousseau had during life several attacks of insanity (Möbius), the poet Cowper suffered from melancholia with delusions, the physiologist Albrecht von Haller became a melancholiac, and Johannes Müller committed suicide; Auguste Comte was subject to attacks of *folie periodique*, made suicidal attempts, and was afflicted in his later days with erotomania and visual hallucinations. Among musical composers Schumann and Donizetti became insane. Among painters Landseer and Munkacszy developed general paralysis of the insane, while Wiertz and Carlo Dolce were insane. William Blake, poet and painter, undoubtedly suffered from insanity with visual hallucinations, and was a seer of visions; while Swedenborg suffered similarly from the auditory and visual hallucinations characteristic of paranoia religiosa. The taint of insanity may thus be present in association with great intellectual powers, and even with genius. Yet the correct way of regarding the subject would be to look upon such taint as a complication, as something superadded. If all forms of nervous trouble occurring in men of genius and of talent be regarded, it will be found, says Féré, that hypochondria is the one most frequently met with. In proof he cites the names of Camoens, Byron, Huyghens, Molière, J. J. Rousseau, Swift, Gilbert, Mozart, Beethoven, &c. A man of genius might equally suffer from headache, or asthma, or migraine. Yet these abnormalities do not constitute an essential or fundamental element in genius. They may be present as collateral complications. It is, indeed, a fact that many men of great talents belong to families with hereditary neuropathic or psychopathic taints, and may have brothers or sisters mentally afflicted. A want of fecundity has also been noticed in men of genius who have married, and occasionally feeble-minded children have been met with among their offspring. On the other hand, genius may be hereditarily transmitted, as shown by Galton in his work on "Hereditary Genius." There are, however, points which are as yet difficult to explain regarding the hereditary transmission of genius. "The average man of genius," says Havelock Ellis,[43] "is short and large-brained . . . . and his general facial expression, as well as his temperament, recall the child." The short stature is ingeniously explained by Mercier in his "Sanity and Insanity" (pp. 173-4). In nearly every department of life, intellectual, ethical, or social, genius has been met with far more frequently among men than among women. The statement of this fact has sometimes been regarded as a slur, or has been met with the answer that lack of opportunity, of education, &c., is the explanation. Yet it is equally true that idiocy, imbecility, and paranoia are also commoner among men. "Genius is more common among men by virtue of the same general tendency by which idiocy is more common among them. The two facts are but two aspects of a larger zoological fact—the greater variability of the male."[44]

The artistic temperament, with its emotional and impulsive tendencies, may be largely developed in some men of genius. An ill-controlled tendency to crimes of passion may be often associated with such a temperament. This, however, should not lead us to accept a statement made by some authors that genius has affinities with crime, as it is said also to have with insanity. It is easy to overstate such resemblances, and to draw conclusions which subsequent and deeper research fails to confirm. While much interesting matter is found in the works of Nisbet[45] and Max Nordau,[46] the facts must be sifted, and the conclusions drawn with more caution than is done by these somewhat popular writers who find only too readily traces of madness or of criminality in men of genius.

[38] Betrachtungen über den Menschen. [39] Psychologie des grands hommes, in Revue Philosophique. [40] Physician and Surgeon, May, 1900. [41] La Psychologie Morbide, Paris, 1859. [42] The Man of Genius, Contemporary Science Series, London. [43] Man and Woman, p. 391. [44] Ibid, p. 366. [45] The Insanity of Genius, London, 1891. [46] Degeneration.

